# Major influence of repetitive elements on disease-associated copy number variants (CNVs)

**DOI:** 10.1186/s40246-016-0088-9

**Published:** 2016-09-23

**Authors:** Ana R. Cardoso, Manuela Oliveira, Antonio Amorim, Luisa Azevedo

**Affiliations:** 1 Instituto de Investigação e Inovação em Saúde, Universidade do Porto, Rua Alfredo Allen 208, 4200-135 Porto, Portugal; 2 IPATIMUP-Institute of Molecular Pathology and Immunology, University of Porto, Rua Júlio Amaral de Carvalho 45, 4200-135 Porto, Portugal; 3 Department of Biology, Faculty of Sciences, University of Porto, Rua do Campo Alegre S/N, 4169-007 Porto, Portugal

**Keywords:** Copy number variants (CNVs), Genetic diseases, Genomic structural variation, Low copy repeats, Retrotransposons, LINE, SINE, Non-allelic homologous recombination (NAHR)

## Abstract

Copy number variants (CNVs) are important contributors to the human pathogenic genetic diversity as demonstrated by a number of cases reported in the literature. The high homology between repetitive elements may guide genomic stability which will give rise to CNVs either by non-allelic homologous recombination (NAHR) or non-homologous end joining (NHEJ). Here, we present a short guide based on previously documented cases of disease-associated CNVs in order to provide a general view on the impact of repeated elements on the stability of the genomic sequence and consequently in the origin of the human pathogenic variome.

## Background

Copy number variants (CNVs) are structural genomic markers (insertions or deletions) ranging in size from 1 kb to several megabytes for each copy. They are categorized as copy number polymorphisms (CNPs) when multiple allelic states exist in the population or as rare copy number variants when they are found to be associated with genetic diseases (pathogenic copy number variants) [1, 2]. The origin of each repeated element of the CNV is influenced by the local genomic architecture which includes the presence of repetitive sequences within or flanking the repeated segment [3–7]. These repeated sequences drive non-allelic homologous recombination (NAHR) events which result in recurrent insertions and deletions with similar sequence sizes and clustered breakpoints [3, 6, 8] or non-homologous end joining (NHEJ) events that result in non-recurrent rearrangements that vary in terms of their size and breakpoint location [3, 6, 9]. Although several studies have been demonstrating the contribution of structural variants to the genome architecture, few have specifically focused the influence of repeated sequences at breakpoint locations. With the aim to draw attention to these unstable regions and to establish their role in CNVs, we collated a number of cases of CNV-associated disorders proven to have been generated by low and high copy number repeats which may have influenced the degree of stability of the genomic sequence.

## Low copy repeats and their influence on pathogenic CNV formation

**Fig. 1:**
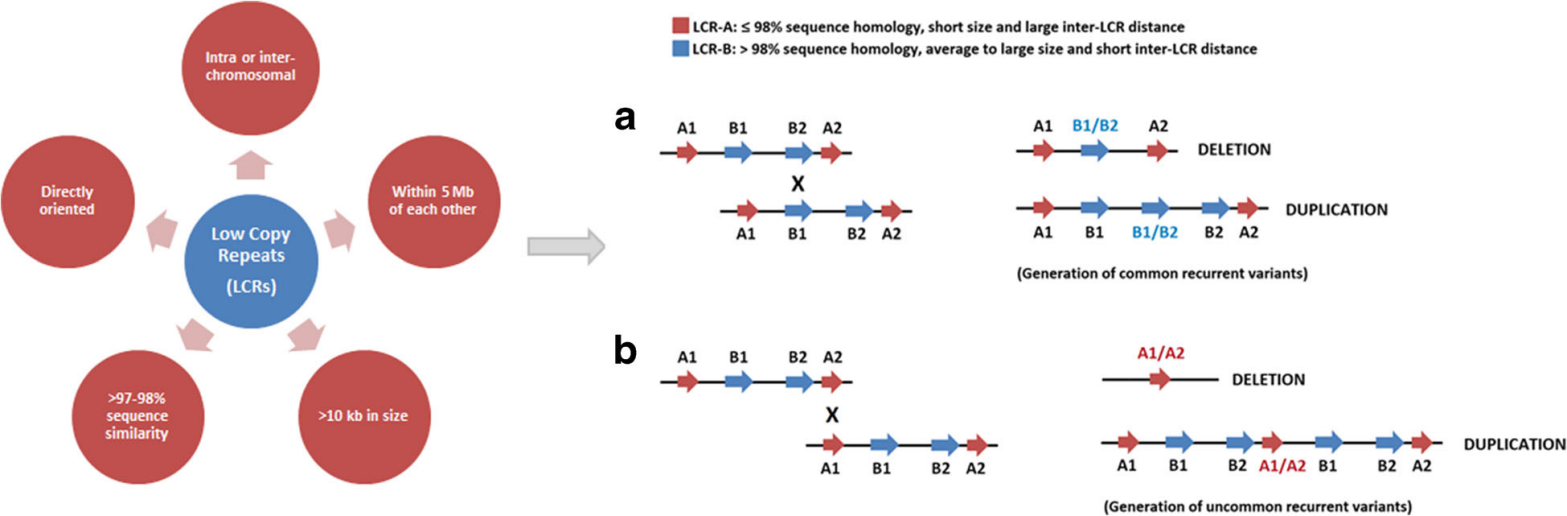
Optimal LCRs features for the occurrence of NAHR events that result in CNV formation. Distinct LCR pairs with counter features such as homology, size, and inter-LCR distance influence NAHR rate and lead to the formation of common recurrent (**a**) or rare recurrent (**b**) copy number variants. Adapted from [3, 6, 12]

Low copy repeats (LCRs) are homologous sequences of ≥1 kb in length which are found in many copies throughout the genome since they are generated by duplication events [3, 10]. Large LCRs (>10 kb) with high sequence homology promote non-allelic homologous recombination (NAHR) [3–6, 10–12] and the misalignment of directly oriented sister chromatids carrying the LCR may promoted NAHR thereby generating both duplications and deletions [4, 5] which in turn give rise to copy number variation. A schematic representation of this process is shown in Fig. 1.

Certain properties of the LCRs such as homology length, sequence similarity, and distance, serve to influence the frequency of NAHR events [3, 6, 12] (Fig. 1). As recently reviewed by Carvalho and Lupski [3], the NAHR rate varies according to the length of the LCR sequence, the distance between distinct LCR sequences and the DNA sequence. The NAHR rate is, therefore, positively correlated with the LCR length but is inversely proportional to the distance between distinct LCRs [3, 9]. Since there is a high homology between distinct LCR sequences proximal to copy number variation regions; there is also an increased predisposition to NAHR events in these genomic regions [3, 4, 6, 9, 12].

A considerable number of disease-associated CNVs generated by LCRs have been documented and reviewed in previous works (e.g. [3, 6]), but for the purposes of this paper, we have only collated cases for which the specific repetitive element was found at the breakpoints of the structural variant and not those for which the causality of the repeats elements was only suggested. The resulting set is presented in Table 1. For example, a complex array of LCRs spanning a 4-Mb region around the X-linked *MECP2* gene was associated with unique duplications ranging in size from 200 kb to 2.2 Mb in developmentally delayed males [13]. Duplications and deletions affecting the *PLP1* gene causing Pelizaeus-Merzbacher disease (OMIM #312080) are also associated with a specific LCR (LCR-PMD A/B pair) within a 3-Mb region flanking the gene in which a multitude of LCRs are located [14]. LCRs are also frequent at the 2q11-q21.1 locus [11], where recurrent deletions of the *NPHP1* gene (2q13) have been associated with nephronophthisis 1 (OMIM #256100). A 0.3-Mb copy number gain was detected in three X-linked intellectual disability (XLID) families and one sporadic patient [15]. The region overlapped the *GDI1* gene, an important XLID-associated gene highly expressed in the brain. The aberration was located in Xq28, a locus that includes other intellectual disability genes and that is frequently associated with recombination events caused by proximal LCRs (e.g., LCR K1/L2). The Angelman syndrome (AS) (OMIM #105830) and Prader-Willi syndrome (PWS) (OMIM #176270) are caused by recurrent 4-Mb deletions at the 15q11-q13 locus. The deleted region is flanked by LCRs [6] and accounts for 70 % of cases of AS and 70–75 % of cases of PWS [16]. The Smith-Magenis syndrome (SMS) (OMIM #182290) results from recurrent deletions of 3.7 Mb at 17p11.2 which account for more than 70 % of cases; about 25 % of affected individuals harbor deletions ranging from 1.5–9 Mb [6, 17]. The deletions are flanked by 200-kb highly homologous LCRs that play a role in generating meiotic NAHR events [16]. These deletions encompass the *RAI1* gene, which is critical in organ and neuronal development—patients with larger deletions manifest a more severe phenotype when the dosage-sensitive gene *PMP22* is deleted [18].

**Table 1:** Repetitive elements detected at the breakpoints of CNVs associated with clinical phenotypes

Phenotype	Critical genes	Type of variant	Locus	Repetitive element involved	Ref.
MECP2 duplication syndrome	*MECP2, L1CAM*	Dup	Xq28	Several LCR-MECP2s pairs	[3, 6, 13, 44–46]
Rett syndrome	*MECP2*	Del	Xq28	Several LCR-MECP2 pairs	[6]
Neurofibromatosis type I	*NF1*	Del	17q11.2	NF1-REPs A/B/C	[3, 6]
Nephronophthisis	*NPHP1*	Del	2q13	Several LCR pairs	[11, 47–49]
Mental retardation, X-linked 41 (MRX41)	*GDI1*	Dup/Trip	Xq28	LCR-K1/L2 pair	[15]
Angelman and Prader-Willi syndromes	*UBE3A*	Del	15q11-q13	END-repeats (LCRs)	[6, 50]
Smith-Magenis syndrome	*RAI1* and *PMP22*	Del	17p11.2	SMS-REPs (LCRs)	[3, 6, 18]
Williams-Beuren syndrome	28 dosage-sensitive genes	Dup/Tripe/Del	7q11.23	A/B/C LCR blocks	[3, 6, 51]
15q13.1 microdeletion syndrome	*CHRNA7*	Dup/Trip	15q13.3	BP3/4/5	[3, 6, 52, 53]
3q29 microduplication or microdeletion syndrome	*DLG1, PAK2*	Dup/Del	3q29	A/B/C LCR blocks	[3, 54, 55]
Pelizaeus-Merzbacher disease	*PLP1*	Dup/Del	Xq22	LCR-PMD A/B pair	[3, 6, 14]
DiGeorge syndrome/velo-cardio facial syndrome	*COMT, TBX1*	Del	22q11.2	8 specific LCR22 repeats	[6, 17, 40]
Charcot-Marie-Tooth type 1A	*PMP22*	Dup	17p12	CMTA1-Reps (LCRs)	[3, 6, 37, 38]

## Retrotransposons (high copy repeats) and their influence on pathogenic CNVs

Interspersed repeats are the most common type of high copy repeats, covering about 44 % of the human genome [4]. Retrotransposons account for the majority of transposable elements [5, 7, 19]. These are mobile elements that through reverse transcription have the ability to integrate into different regions [7, 19]. Long interspersed nuclear elements (LINEs), short interspersed nuclear elements (SINEs), and retrovirus-like elements (LTR transposons) are the three major categories of mammalian retrotransposons (Table 2).

**Table 2:** Main characteristics of the most abundant retrotransposons [4, 5, 7, 19]

	Retrotransposons (interspersed repeats)—44 % human genome		
	Non-long terminal repeat (LTR)		Long terminal repeat (LTR)
Repetitive element	Long interspersed nuclear repeats (LINEs)	Short interspersed nuclear repeats (SINEs)	Endogenous retroviruses (ERV)
Genomic coverage	20 %	11 %	8 %
Features	• L1 is the most abundant class • Autonomous transposons • Reverse transcriptase (RT) encoded by LINE-1	• Alu is the most abundant class • Dependent on LINEs transposable machinery • Mobile polymerase III promoter • 100–400 bp in length	• Reduced transposable activity • Presence of *gag* and *pol* viral genes

Among LINEs, L1 is the most abundant element, typically of 6–8 kb in length, with the ability to increase genomic instability through NAHR events [4]. It is known that about 83 % of the human genome is prone to LINE-LINE recombination events that contribute to genomic instability and can give rise to unbalanced structural variants [20].

Alu elements are the most common SINEs and have been associated with NAHR events that lead to pathogenic duplications and deletions [3, 4, 21, 22]. Table 3 presents examples of high copy repeats that have been detected at the breakpoints of disease-associated CNVs.

**Table 3:** High copy repeats detected at the breakpoints of CNVs associated with clinical phenotypes

Phenotype	Critical genes	Type of variant	Locus	Repetitive elements involved	Ref.
Peutz-Jeghers syndrome	*STK11*	Del	19p13.3	Several AluY/AluY pairs	[23]
Spastic paraplegia 4	*SPAST, SLC30A6*	Dup/Del	2p22.3	Several Alu pairs	[25]
OTC deficiency	*OTC*	Del	Xp11.4	AluSx/AluSq pair	[27, 56]
Miller-Dieker syndrome and 17p13.3 duplication syndrome	*LIS1*	Del	17p13.3	Several Alu pairs	[6, 24]
Breast cancer	*BRCA1*	Del	17q21.31	AluSx/AluSc pair	[29, 57]
Autosomal dominant adult-onset demyelinating leukodystrophy (ADLD)	*LMNB1*	Dup/Trip	5q23.2	LIPA3 LINE repeats	[26]
				AluYA/AluYB pair	
Azoospermia	*AZFa*	Del	Yq11	HERV15 A/B proviruses	[34, 58]
Mental retardation, X-linked 60 (MRX60)	*OPHN1*	Del	Xq12	AluY/AluY pair	[35]
Pelizaeus-Merzbacher disease	*PLP1*	Del	Xq22	AluSq/AluSx pair	[3, 6, 59]
DiGeorge syndrome/velo-cardio facial syndrome	*COMT, TBX1*	Del	22q11.2	Unclassified Alu/Alu pair	[6, 17, 40]
Charcot-Marie-Tooth type 1A	*PMP22*	Dup	17p12	AluY/AluY pair	[39]
				AluSg/AluSg pair	
Williams-Beuren syndrome	28 dosage-sensitive genes	Dup/Del	7q11.23	AluS subfamily elements	[36]
Parkinson’s disease	*SNCA*	Dup/Trip	4q21	Several Alu pairs	[32]

Borun and colleagues [23] reported the presence of CNV breakpoints within Alu elements in the *STK11* gene which lead to the Peutz-Jeghers syndrome (OMIM #175200), where CNVs account for 30 % of cases. The 17p13.3 locus is enriched in copy number variations associated with genomic disorders, such as the Miller-Dieker syndrome (17p13.3 deletion syndrome) (OMIM #247200) and its reciprocal 17p13.3 duplication syndrome (OMIM #613215) [24]. The breakpoints of the reported CNVs at this locus are highly enriched in Alu elements, which mediate these junctions through an Alu-Alu mechanism. About 70 % of CNVs found in the *SPAST* gene have been associated with Alu recombination events [25]. Local Alu-rich architecture predisposes to the formation of pathogenic structural rearrangements associated with spastic paraplegia (OMIM #182601). An extra copy of the *LMNB1* gene at the 5q23 locus has been previously associated with autosomal dominant adult-onset demyelinating leukodystrophy (ADLD) (OMIM #169500). The analysis of twenty ADLD-affected families revealed sixteen duplications ranging from 128 to 475 kb in size, all of them spanning the *LMNB1* gene [26]. The centromeric region of the critical gene is enriched with SINE elements, particularly Alus. Alu-mediated recombination events were also found to be linked to pathogenic deletions at the *OTC* gene [27], a urea cycle gene for which a significant number of structural variants are known [28]. NAHR events between Alu repeats are also strongly correlated with the birth of structural rearrangements at the Alu-rich *BRCA1* locus [29] which is associated with breast cancer. Duplications (220 to 394 kb) and a triplication (1.61 to 2.04 Mb) of the *SNCA* gene located at 4q21 locus have been implicated in autosomal dominant Parkinson’s disease (PD1 and PD4) (OMIM #168601, #605543). The phenotypic severity is consistent with a gene dosage effect [6]. Regarding recessive PD (OMIM #600116), about one third of pathogenic variants associated with the *PRKN* gene are CNVs occurring between exon 2 and exon 5, which may therefore be considered to be a recombination hotspot [30, 31]. Ross and colleagues [32] reported the presence of Alu and LINE1 elements at the *SNCA* locus that may contribute to the genomic instability at this locus.

Human endogenous retroviruses (HERVs) represent about 4.9 % of the human genome [4]. Sequences with about 95 % sequence similarity were previously associated with NAHR events and recurrent CNVs, some of which with pathogenic implications [3, 33]. For example, the occurrence of NAHR between a particular set of HERV elements flanking the male fertility *AZFa* locus in the Y chromosome is strongly associated with pathogenic deletions associated with male infertility (OMIM #400042, #415000) [4, 34].

## Pathogenic copy number variants associated with both LCRs and retrotransposons

The breakpoints of some disease-associated CNVs have been reported to be caused by more than one type of repetitive elements which indicates that the same phenotype involves both low copy and high copy repeats that affect the stability of a target gene. Bergmann and colleagues [35] conducted a family study in which five brothers shared the same phenotypic pattern that included intellectual disability. The analysis of the OPHN1 locus (Xq12) revealed the presence of a 17.6-kb intronic deletion and the breakpoints spanning the deletion revealed two highly homologous Alu repeats and additional repetitive sequences (interspersed and simple repeats).

A recurrent deletion of 1.6 to 1.8 Mb (>95 % of the patients) at the 7q11.23 locus causes the Williams-Beuren syndrome (OMIM #194050) [6]. Genes within this region are dosage-sensitive and the recurrently deleted region encompasses a total of 28 genes. This locus is characterized by highly homologous flanking LCRs that contribute to NAHR events [6]. Antonell and colleagues [36] reported the presence of Alu elements at the junctions of large duplicated blocks in 7q11.23 suggesting the influence of these retrotransposons in the generation of large LCRs.

Heterozygous duplication and reciprocal deletions of a 1.4–1.5-Mb segment at the 17p12 locus have been previously linked with the Charcot-Marie-Tooth type 1A syndrome (CMT1A) (OMIM #118220). About 70 % of CMT1A patients have a recurrent duplication of the dosage-sensitive PMP22 locus and the NAHR event that gave rise to this copy number variation was mediated by LCRs [3, 6, 37, 38]. A study by Zhang and colleagues [39] revealed the presence of SINEs (Alu elements) and LINEs (L1 and L2) as well as LCRs within the breakpoints of rare non-recurrent deletions and duplications at the CMT1A locus.

About 96 % of the DiGeorge syndrome (DGS) (OMIM #188400)- and velo-cardio-facial syndrome (VCFS) (OMIM #192430)-affected patients harbor a 1.5–3 Mb deletion at the 22q11.2 locus that includes 24 to 30 genes [16]. The breakpoints of the common recurrent deletions at this locus are associated with LCRs [17] and one Alu sequence [40]. Both the deletions and duplications at this locus are generated by NAHR events between the repeated regions flanking the CNV, specifically the low copy repeat known as LCR22 [41]. Furthermore, 20–25 % of individuals who harbor this deletion also show signs of schizophrenia, mood disorders, and other behavioral alterations [41].

## Conclusions

Although the majority of genetic diseases are caused by non-structural variants (e.g. [42, 43], an increasing number of causative mutations have been associated with CNVs and these cases were the focus of this short review. Low copy repeats and retrotransposons are the major contributors to CNV formation. Recurrent CNVs are mainly directed by NAHR events that occur between highly homologous LCR sequences. In terms of non-recurrent CNVs, NHEJ (among other molecular mechanisms [3]) generally occurs between sequences with a degree of homology lower than that observed between distinct LCRs. The diversity of breakpoint junctions of non-recurrent variants renders the establishment of phenotype-genotype relationships less reliable because the sequence that is deleted or duplicated in each patient is different and the affected region may also involve other genes. This review focused on disease-associated CNVs in order to show that although numerous cases of instability driven by repeated sequences around the affected locus (or loci) have been documented, we are still far from understanding all the phenotypic complexities associated with these unbalanced variants, mainly because the number of reported cases is still too small to draw general conclusions. Finally, it is important to mention that collated data, such as those presented in this paper, pertaining to the pathogenic structural variome are expected to drive future studies with the aim of establishing a map of unstable genomic hotspots which promises to be useful in the context of clinical genetic testing where the determination of the molecular basis of Mendelian and complex diseases (e.g., cancer) is of paramount importance.

## Abbreviations

CNV: Copy number variant
LCR: Low copy repeat
CNP: Copy number polymorphism
LINE: Long interspersed nuclear repeat
SINE: Short interspersed nuclear repeat
LTR: Long terminal repeat
NAHR: Non-allelic homologous recombination
NHEJ: Non-homologous end joining
XLID: X-linked intellectual disability
